# Automated segmentation of endometrial cancer on MR images using deep learning

**DOI:** 10.1038/s41598-020-80068-9

**Published:** 2021-01-08

**Authors:** Erlend Hodneland, Julie A. Dybvik, Kari S. Wagner-Larsen, Veronika Šoltészová, Antonella Z. Munthe-Kaas, Kristine E. Fasmer, Camilla Krakstad, Arvid Lundervold, Alexander S. Lundervold, Øyvind Salvesen, Bradley J. Erickson, Ingfrid Haldorsen

**Affiliations:** 1 NORCE Norwegian Research Centre, Bergen, Norway; 2 Department of Radiology, MMIV Mohn Medical Imaging and Visualization Centre, Haukeland University Hospital, Bergen, Norway; 3 Section for Radiology, Department of Clinical Medicine, University of Bergen, Bergen, Norway; 4 Department of Mathematics, University of Bergen, Bergen, Norway; 5 Department of Clinical Science, Centre for Cancer Biomarkers, University of Bergen, Bergen, Norway; 6 Department of Obstetrics and Gynecology, Haukeland University Hospital, Bergen, Norway; 7 Department of Biomedicine, University of Bergen, Bergen, Norway; 8 Western Norway University of Applied Sciences, Bergen, Norway; 9 Department of Public Health and General Practice, Norwegian University of Science and Technology, Trondheim, Norway; 10 Department of Radiology, Mayo Clinic, Rochester, MN USA

**Keywords:** Cancer imaging, Computational science

## Abstract

Preoperative MR imaging in endometrial cancer patients provides valuable information on local tumor extent, which routinely guides choice of surgical procedure and adjuvant therapy. Furthermore, whole-volume tumor analyses of MR images may provide radiomic tumor signatures potentially relevant for better individualization and optimization of treatment. We apply a convolutional neural network for automatic tumor segmentation in endometrial cancer patients, enabling automated extraction of tumor texture parameters and tumor volume. The network was trained, validated and tested on a cohort of 139 endometrial cancer patients based on preoperative pelvic imaging. The algorithm was able to retrieve tumor volumes comparable to human expert level (likelihood-ratio test, $p = 0.06$). The network was also able to provide a set of segmentation masks with human agreement not different from inter-rater agreement of human experts (Wilcoxon signed rank test, $p=0.08$, $p=0.60$, and $p=0.05$). An automatic tool for tumor segmentation in endometrial cancer patients enables automated extraction of tumor volume and whole-volume tumor texture features. This approach represents a promising method for automatic radiomic tumor profiling with potential relevance for better prognostication and individualization of therapeutic strategy in endometrial cancer.

## Introduction

Endometrial cancer (EC) is the most common gynecologic cancer in industrialized countries^1^. EC is surgicopathologically staged according to the International Federation of Gynecology and Obstetrics (FIGO) staging system. Most patients present with early-stage disease are cured by surgery; however, about 15% develop recurrence with limited treatment options and poor survival^2^. Routinely reported preoperative imaging findings guide surgical approach, and advanced imaging markers may also enable more individualized surgical and adjuvant treatment strategies. Valid identification of high-risk patients who are likely to profit from the most invasive surgical procedures or from adjuvant therapy, is necessary in order to improve clinical patient care. Thus, novel imaging analyses yielding better tools for refined preoperative risk-stratification are urgently needed.

A large family of image texture parameters has been shown to predict high-risk disease and reduced disease specific survival in EC^3–6^. Also, large primary tumor volume is known to predict advanced stage and aggressive disease^7^. Manual whole-volume tumor segmentation for accurate calculation of tumor volumes and for generation of whole-volume texture features is, however, very labor intensive and thus not feasible in daily clinical routine. Thus, automatic methods for segmentation of tumors in EC are needed. After the success of ImageNet in the Large Scale Visual Recognition Challenge^8^, deep learning and convolutional neural networks (CNNs) have been increasingly utilized to solve various classification problems in computer vision across domains, also within diagnostics and medicine^9,10^, e.g. for skin lesion classification^11,12^, for identification of intracranial hemorrhages based on CT scans^13^, for assessing mortality from chest radiographs^14,15^, and for histologic classification in breast cancer^16^.

To date, the majority of deep learning applications have been tailored for segmenting tumors on two-dimensional images, with a large number of applications using variants of the two-dimensional U-Net convolutional neural network architecture introduced previously^17^. However, high-resolution 3D imaging modalities like MRI are commonly used at primary diagnosis for treatment planning in cancer. Recently, platforms and powerful frameworks readily available for implementation of medical imaging-based deep-learning applications in 3D have been introduced^18–21^. This study aimed to utilize 3D CNNs to develop a fully automatic system for the detection and delineation of primary EC tumors in 3D in order to facilitate automated extraction of whole-volume tumor texture features and tumor volumes.

## Results

**Figure 1:**
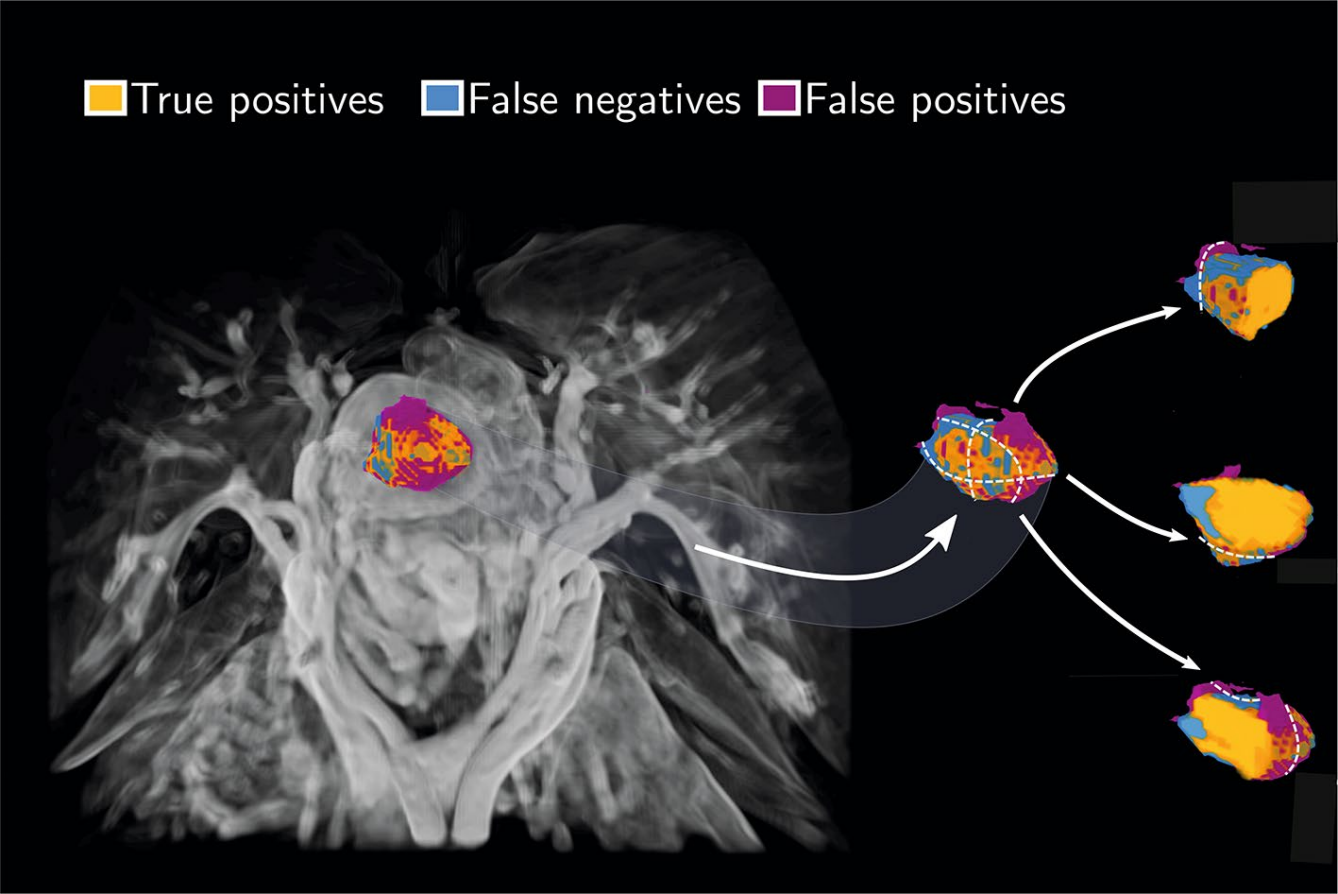
Volume rendering (left panel) of manual and automated ML segmentation of the primary tumor from one patient in terms of true positives (orange), false negatives (blue), and false positives (purple). The 3D segmentation is shown along with the gradient-enhanced 3D volume rendering of the T1-weighted VIBE sequence as a background image. Furthermore, three separate paraxial slices from the same data set are depicted together with a close-up view of the tumor and outlined segmentations (right panel). A majority of the false predictions occur in the tumor periphery, indicated with blue and purple labels. The white, dotted lines are the three orthogonal cutting planes used for creating the corresponding slicing plot.

**Figure 2:**
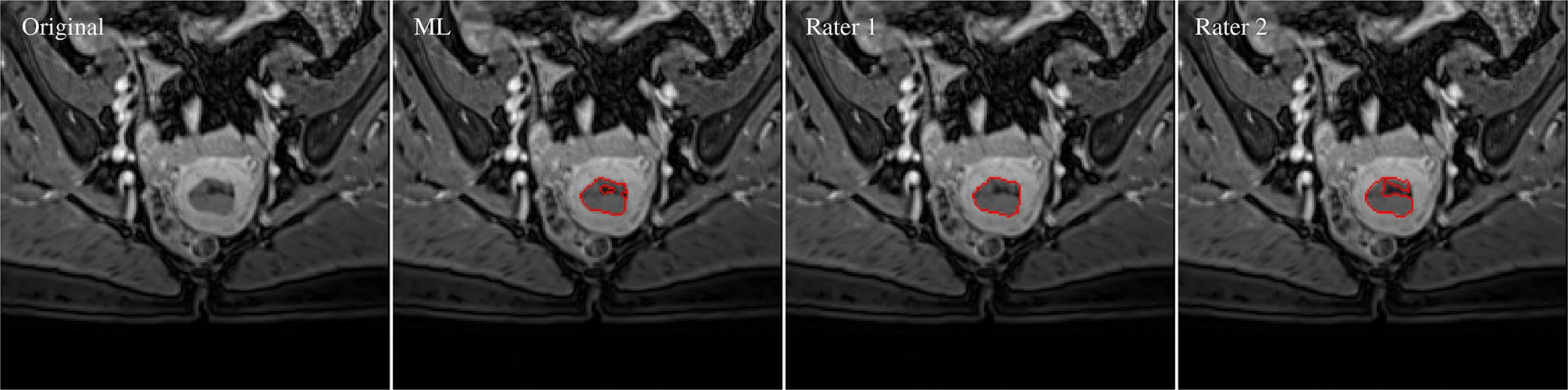
Tumor contours of machine learning and the two raters (red solid curves). The contours are superimposed on the VIBE image. From left to right: Original VIBE image, contours of ML, rater 1 (R1) and rater 2 (R2). Dice similarity coefficients of the respective comparisons are reported as DSC$_{\text {ML,R1}}=0.96$, DSC$_{\text {ML,R2}} = 0.89$, DSC$_{\text {R1,R2}}=0.90$ respectively.

A total of $n=34$ patients comprising the test dataset were used for evaluation of performance. A 3D volume rendering of the machine learning (ML) segmentation aligned with the tumor segmentation of rater 1 for one of the patients in the validation data set is shown in Fig. 1. The figure depicts large regions of agreement in terms of true positives, and also regions of disagreement in terms of false positives and negatives. Examples of 2D contours taken from the 3D segmentation masks of machine learning and of the two raters are shown in Fig. 2.

### CNN-based tumor volume estimation

**Figure 3:**
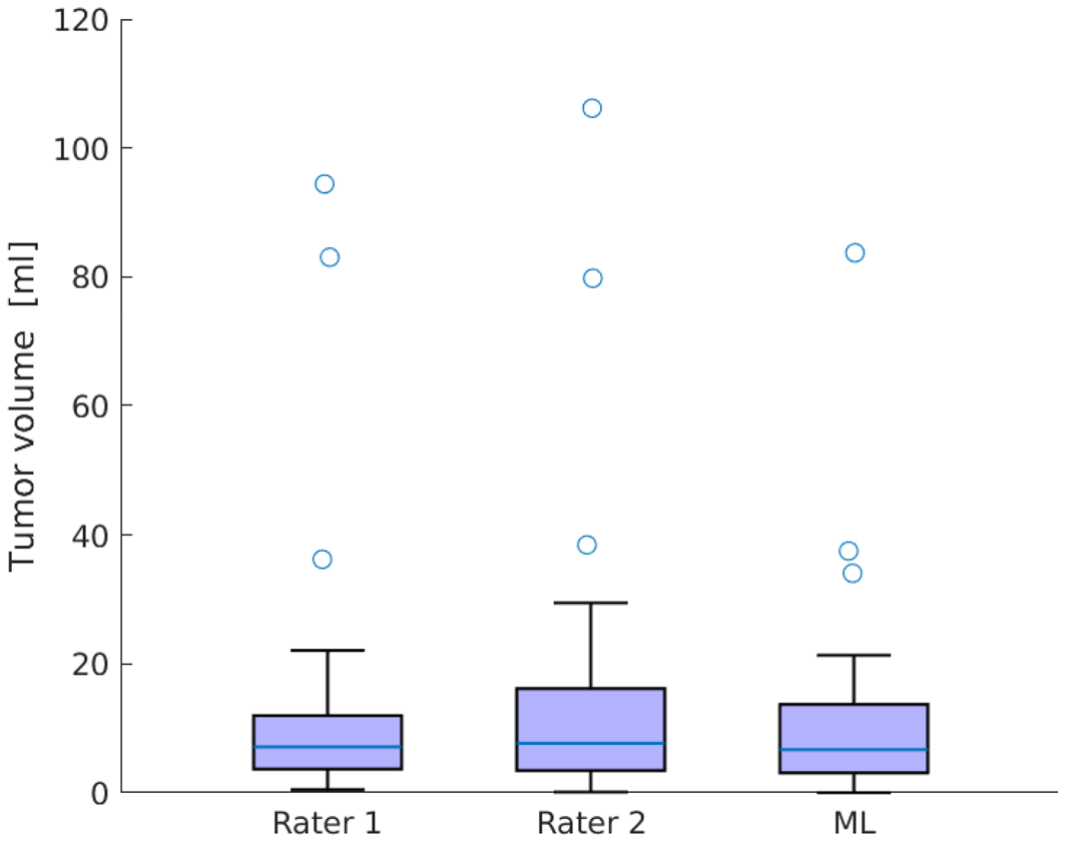
Tumor volumes of rater 1, rater 2, and machine learning. We found no difference in median tumor volume between the raters and the machine learning application (Friedman test, $p = 0.28$). For each box, the central mark shows the median, and the box extends to the 25th and 75th percentiles. The whiskers extend to the most extreme data not considered outliers, while outliers are plotted individually.

We found no difference in median tumor volume between the two raters and the machine learning application (Friedman test, $p = 0.28$). A box plot of tumor volumes is shown in Fig. 3.

**Figure 4:**
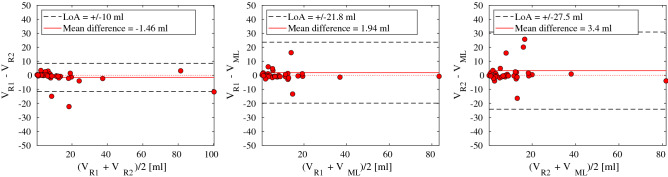
Bland-Altman plots of estimated primary tumor volume [ml] between raters and machine learning. Left: Rater 1 tumor volume ($V_{R1}$) compared to rater 2 tumor volume ($V_{R2}$). Middle: Rater 1 tumor volume ($V_{R1}$) compared to machine learning tumor volume ($V_{ML}$). Right: Rater 2 tumor volume ($V_{R2}$) compared to machine learning tumor volume ($V_{ML}$). Dashed lines indicate limits of agreement (LoA), and the solid red line represents the mean difference.

We found high agreement in tumor volume between the two raters, suggesting that 95% of the observations of expert rater 2 will lie within an interval of $+/-$ 10 ml with a bias of − 1.46 ml to expert rater 1 (Bland-Altman plot in Fig. 4 , left panel). Strong agreements were also found between the raters and the machine learning (Bland-Altman plot in Fig. 4, middle and right panels). These plots suggest that 95% of the observations of the machine learning segmentation will stay within an interval of $+/-$ 21.8 ml with a bias of 1.94 ml from rater 1, and within $+/-$ 27.5 ml with a bias of 3.4 ml from rater 2.

Excellent consistency in log tumor volume was found between the two raters (ICC$_{\text {R1,R2}}$ = 0.86, 95% CI = (0.77, 0.94), CI = confidence interval). A high consistency was also found between the machine learning with any of the raters (ICC$_{\text {R,ML}}$ = 0.76, 95% CI = (0.64, 0.89)). The intraclass correlation coefficients ICC$_{\text {R1,R2}}$ and ICC$_{\text {R,ML}}$ were not statistically different from each other (likelihood-ratio test, $p=0.06$), suggesting that the agreement in tumor volume is comparable between humans and between humans and machine learning.

### Evaluation of volumetric segmentation in terms of Dice coefficients, Hausdorff distances, and average surface distance

**Figure 5:**
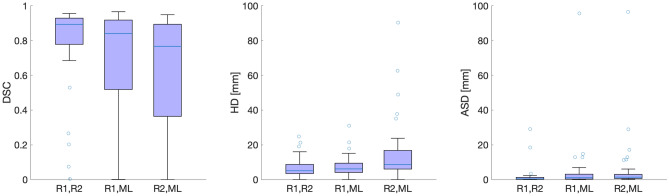
Box plot of DSC, HD, and ASD obtained when comparing segmentations between raters and ML. Left plot: Median DSC obtained for inter-rater agreement (left box: R1, R2) is higher than between raters and ML (middle and right boxes). Middle plot: The lowest median HD was obtained for inter-rater agreement (left box: R1, R2), followed by ML with both of the raters (middle and right boxes). Right plot: The lowest median ASD was obtained for inter-rater agreement (left box: R1, R2), followed by ML with both of the raters (middle and right boxes). R = rater.

Median DSC for rater 1 with rater 2, rater 1 with ML, and rater 2 with ML were 0.89, 0.84 and 0.77, respectively (cfr. Fig. 5, left panel). In the plot, five outliers are seen (open dots), representing cases with particularly high disagreement between raters. Median tumor volume in the outliers (5.44 ml) was not different from median volume in the remaining tumors (7.65 ml) (Wilcoxon rank sum test, $p=0.43$). Median values of HD were 5.15 mm for rater 1 with rater 2, 6.39  mm for rater 1 with ML and 8.69 mm rater 2 with ML (Fig. 5, middle panel). Median values of ASD were 0.43 mm for rater 1 with rater 2, 1.08 mm for rater 1 with ML and 1.43 mm rater 2 with ML (Fig. 5, right panel).

**Table 1:** Differences in DSC, HD, and ASD between human raters (R1, R2) and machine learning (ML) in the test cohort.

I. Median DSC, HD, and ASD					II. Statistical comparison	
Parameter	Subtype	A (R1 vs. R2)	B (R1 vs. ML)	C (R2 vs. ML)	|A–B| (p)	|A–C| (p)
DSC	All patients	0.89	0.84	0.77	0.05 (0.08)	0.12 (< **0.001**)
	Endometroid	0.89	0.86	0.81	0.03 (0.12)	0.08 (<** 0.001**)
	Non-endometroid	0.90	0.52	0.48	0.38 (0.44)	0.41 (0.13)
HD (mm)	All patients	5.15	6.40	8.69	1.25 (0.60)	3.54 (**0.0072**)
	Endometroid	5.10	5.83	7.07	0.73 (0.93)	1.97 (<** 0.001**)
	Non-endometroid	3.46	9.43	10.0	5.97 (0.06)	6.54 (0.13)
ASD (mm)	All patients	0.42	1.08	1.43	0.66 (0.05)	1.00 (<** 0.001**)
	Endometroid	0.43	0.98	1.06	0.55 (0.11)	0.63 (**0.005**)
	Non-endometroid	0.40	6.10	6.09	5.70 (0.55)	5.68 (0.31)

Performance is reported for all patients and separately for endometroid ($n=30$) and non-endometroid ($n=5$) subtypes. **I**: Median value of agreements A = rater 1 with rater 2 (inter-rater agreement), B = rater 1 with ML, C = rater 2 with ML of DSC, HD, and ASD. **II**: Absolute difference in median value (p-values in brackets) of the comparisons A with B and A with C, corresponding to inter-human agreement compared to human with ML. Significant differences are marked with bold. For all patients there was no difference in inter-rater agreement compared with ML against rater 1 for DSC, HD, or ASD (A–B: p = 0.08, p = 0.60, and p = 0.05, respectively) whereas lower agreement was observed between rater 2 and ML compared to inter-rater agreement for DSC, HD, and ASD (A–C p $\le$  0.0072 for all). Similar differences between ML and rater 2 compared to inter-rater agreement were found for the endometroid subtype (p $\le$  0.01), but not for rater 1. No statistical differences in performance were detected for non-endometroid subtype although the difference in estimates |A–B| and |A–C| of DSC and ASD were higher for non-endometroid subtype than for endometroid subtype.

Median DSC was higher for inter-rater agreement than between the raters and ML (Table 1, panel I, DSC=0.89 versus DSC=0.84 and DSC=0.77), with a difference in estimate to the human raters of 0.05 and 0.12, respectively. A similar result was obtained for HD, where the most optimal HD value was obtained for inter-rater agreement (Table 1, panel I, HD = 5.15 mm versus HD = 6.40 mm and HD = 8.69 mm) with absolute differences in estimate to the human raters of 1.25 and 3.54 Also for ASD the most optimal value was obtained for inter-rater agreement (Table 1, panel I, ASD = 0.42 mm versus ASD = 1.08 mm and ASD = 1.43 mm) with absolute differences in estimate to the human raters of 0.66 mm and 1.00 mm.

Pairwise comparisons in terms of DSC and HD revealed that inter-rater agreement was not different to agreement between rater 1 with ML, neither in terms of DSC, HD or ASD (Table 1, panel II, A–B, Wilcoxon signed rank test, p = 0.08, p = 0.60, and p = 0.05, respectively). However, inter-rater agreement was higher than agreement between rater 2 and ML in terms of DSC HD, and ASD (Table 1, panel II, A–C, Wilcoxon signed rank test, p < 0.001, p = 0.0072, and p < 0.001).

Segmentation performance for the endometroid subtype was similar to that of the full cohort, with a statistical difference in performance between inter-rater agreement and between rater 2 and ML, in terms of DSC, HD, and ASD (Table 1, panel II, Endometroid, A–C, Wilcoxon signed rank test, p<0.001, p<0.001, p = 0.005), whereas such a difference was not found for rater 1 and ML compared to inter-rater agreement. No statistical differences on segmentation performance was observed for non-endometroid subtype, although the differences in estimates A–B and A–C of DSC, HD and ASD were larger than for the endometroid subtype.

## Discussion

This study presents a fully automatic approach for segmentation of primary tumor in endometrial cancer using three-dimensional convolutional neural networks applied to preoperative pelvic MR images. By this approach, tumor volume estimates and segmentation accuracy based on CNNs are comparable to manual segmentation by radiologists. Automated and precise tumor segmentation using CNN provides new opportunities for expeditious whole-volume radiomic tumor profiling, potentially yielding prognostic markers that may enable more personalized EC treatment.

We found no difference in median tumor volume between human raters and machine learning (Friedman test, $p=0.28$), suggesting that on average for a larger sample, the machine learning application has no detectable bias. Hence, the automatic segmentation method has potential to reveal systematic changes in tumor volume between groups in response to various treatment strategies, with a high value in clinical drug trials.

In regard to individual tumor volume estimates, we found a very high agreement in log tumor volume between human raters (ICC$_{\text {R1,R2}}$ = 0.86). High agreement in machine learning log tumor volume and any of the raters was also observed (ICC$_{\text {R,ML}}$ = 0.76). Importantly, there was no significant difference in these ICC estimates, suggesting that the network provides a comparable agreement with human experts as between humans.

We also investigated volumetric segmentation accuracy in terms of Dice similarity coefficients (DSC), average surface distance (ASD), and Hausdorff distances (HD).

Notably, the expert raters disagreed substantially on primary tumor placement in five out of 35 patients (cfr. Fig. 5, open dots in left panel). Interestingly, these five cases had similar tumor size to that of the non-outliers (Wilcoxon rank sum test, $p=0.43$); thus, the observed disagreement is not due to smaller tumor size but seems to be due to less obvious factors causing lower agreement for tumor segmentations.

Human agreement compared to CNN showed no differences when network performance was compared to rater 1 in terms of DSC, HD and ASD (Table 1, Wilcoxon signed rank test, $p=0.08$, $p=0.60$, and $p=0.05$ respectively). The absolute difference in estimate compared to inter-rater agreement was DSC = 0.05, HD = 1.25 mm, and ASD = 0.66 mm. However, when comparing CNN performance with rater 2, human agreement was better in terms of all evaluation criteria DSC, HD, and ASD (Wilcoxon signed rank test, p < 0.01) with a median difference in DSC = 0.12, HD = 3.54 mm, and ASD = 1.00 mm.

Similar results showing differences in performance between rater 2 and ML compared to inter-rater agreement were found in the endometroid subtype (Wilcoxon signed rank test, $p<0.005$, with absolute median differences in DSC = 0.08, HD = 1.97 mm, and ASD = 0.63 mm. No differences were found for rater 1. No significant difference in agreement was observed in the non-endometroid subtype although the differences in estimate were larger than in the endometroid group (maximum |A-B| and |A-C|: DSC = 0.41, HD = 6.54 mm, and ASD = 5.70 mm). Notably, the results must be interpreted with care due to the low number of patients with non-endometroid subtype in the test cohort (n = 5).

The differences found between raters and ML probably reflects that the CNN was trained on more labeled training masks from rater 1 (n = 79) than from rater 2 (n = 26). However, our study indicates that even with the relatively small number of training masks provided by rater 1, it was possible to create a CNN yielding high agreement with rater 1, similar to the inter-rater agreement of human experts. Thus, we hypothesize that by including more labeled data sets from rater 2, it is likely that the segmentation network would be able to yield similar agreements between ML and both readers and at the same level as the inter-rater agreement. Thus, we believe that if adding multiple raters who all segment a substantial number of data sets, the network is likely to perform at a comparable level to that of all raters.

Furthermore, we found no differences in Hausdorff distances between inter-rater agreement and ML compared with rater 1. This result points out that the CNN was able to provide tumor masks with predominantly locally extending variations around the tumor in comparison to human experts. This is a highly interesting finding in this study, as it suggests that most over- and under-segmentation errors are localized close to the tumor periphery. Visual inspection of the segmented data sets also confirmed this hypothesis. As such, we have reason to believe that the central tumor areas are most reliably detected by the CNN approach, with a large potential for automatic generation of tumor texture variables that reportedly yield promising imaging biomarkers for classification into low- and high-risk disease.

An important limitation of our study is the relatively small cohort used for training of the CNNs. Hence, a follow-up study should be based on larger multi-center imaging data including heterogeneous data from different vendors, acquisitions, human operators, and patient groups. The study is also limited by the inherent limitations of retrospective as opposed to prospective designs. Accurate manual tumor labeling of 3D image data is highly labor intensive, and future studies on automated EC tumor segmentation should explore whether using novel methodologies for semi-^22^ and partially supervised^23^ learning is able to yield even better segmentation accuracy.

In brief, this study shows that existing machine learning algorithms are able to provide reliable tumor segmentations at human expert level in endometrial cancer patients. Our setup yields tumor volume, tumor boundaries, and volumetric tumor maps. Hence, the automatic approach for EC primary tumor segmentation has the potential to provide close to real-time whole-volume radiomic tumor profiling including tumor volume and tumor texture properties, with potential relevance for risk-stratification and for developing more personalized treatment strategies in endometrial cancer.

## Methods

The current work is a retrospective study of a cohort of 139 endometrial cancer patients undergoing pelvic MR imaging prior to hysterectomy between May 2009 and April 2019^6^. Eligible participants formed a random selection taken from a consecutive series of patients. The patients were diagnosed and treated at Haukeland University Hospital, serving a population of  1 million inhabitants from the Western part of Norway. Inclusion criterion was histologically confirmed endometrial cancer, and diagnostics was confirmed in a routine diagnostic work-up. The study was conducted under approval of REC West, the Regional Committee for Medical and Health Research Ethics with written informed consent from all patients. All experiments and methods were performed in accordance with relevant guidelines and regulations. The patient cohort was previously used in the context of other research questions than segmentation, with a maximum overlap of $n=70$ patients^2,3,6,7^.

Histologic subtype was endometrioid ($n=114$) or non-endometroid ($n=25$) (comprising clear cell, serous papillary, carcinosarcoma, or undifferentiated subtype). The number of patients with endometrioid and non-endometrioid subtype in the train/validation and test cohorts were $85+29$ (endometroid) and $20+5$ (non-endometroid), respectively. Segmentation performance was analyzed separately for the two histologic subtypes.

### MRI protocol

Patients in the cohort underwent preoperative pelvic MRI examinations at Haukeland University Hospital, Bergen, Norway, either on a 1.5T Siemens Avanto MR scanner ($n=71$), or on a 3T Siemens Skyra MR scanner ($n=68$). The imaging sequence (Table 2) used in this study was a contrast enhanced (2 min delay after contrast injection ) T1-weighted axial oblique 3D volumetric interpolated breath-hold (VIBE) gradient echo sequence with fat saturation. DICOM image data were exported to NIfTI-1 data format^24^ employing the DICOM to NIfTI conversion tool mri_convert.

**Table 2:** MR acquisitions used in the project for segmentation of primary tumors in endometrial cancer. deg = degrees, mm = millimeter, ms=millisecond, FA = flip angle, FOV = field of view, PA = paraxial slice orientation, TE = echo time, TR = repetition time, VIBE = Volumetric Interpolated Breath-hold Examination, FS = fat saturated.

MR machine	Sequence	Plane	TR/TE (ms)	FA (deg)	Slice thickness (mm)	Matrix	FOV (mm$^3$)	Voxel size (mm$^3$)
1.5T Siemens Avanto ($n = 71$)	FS T1 VIBE	PA	7.23/2.55	17	2.0	$192\times 192$	$250\times 250\times 96$	$1.30\times 1.30\times 2.00$
3T Siemens Skyra ($n=68$)	T1 VIBE DIXON	PA	5.86/2.46	9	1.2	$160\times 160$	$227\times 250\times 106$	$0.98\times 0.98\times 1.20$

### Deep learning methodology

A 3D convolutional neural network (UNet3D)^21^ using Keras^25^ (https://github.com/keras-team/keras.git) and Tensorflow^26^ as backend engine was used for 3D segmentation of endometrial primary tumors. The core network implementation was retrieved from github https://github.com/ellisdg/3DUnetCNN^21^ and wrapped into a dedicated Python implementation facilitating data augmentation, training, validation, and prediction. A customized image data generator for 3D images was applied for data augmentation (https://github.com/keras-team/keras/blob/master/keras/preprocessing/image.py), including rotation (rotation_range = 0.1), zooming (zoom_range = 0.15), and horizontal flip. For each patient, eight randomly placed window samples were extracted for training. Windows sampled with overlap to the image boundary or background levels were not allowed in order to avoid padding out-of-view data for training. A majority of default parameter settings were applied for training of the CNN. Default parameters are marked with *: Shape of input data = (192, 192, 32) voxels$^3$, depth of the U-shape of the model = 4, initial learning rate* = 1e−5 (learning rate was decayed during training), poolsize for max pooling operations* = (2,2,2), number of base filters in the first layer in the convolution network* = 32, convolution (deconvolution) operations instead of upsampling* = False, maximum number of epochs = 1500, activation function = “sigmoid” , batch normalization* = False, number of labels = 1, metrics* = Dice coefficient, batch size = 5, and optimizer = RMSprop. The training data set was randomly shuffled between epochs.

Exploration and tuning of hyperparameters included modifying the shape of input data, depth of the model, number of base filters, convolution (deconvolution) operations/upsampling, activation function = sigmoid/ReLu, and batch normalization False/True. A train/validation split of 0.2 was applied to the input data of the model. Convergence of the model was monitored on the validation data set with patience = 30 from keras.callbacks.EarlyStopping in order to stop training when there is no further improvement of the model. The machine-learning (ML) network resulted in smooth predictions. A higher prediction value is associated with a higher likelihood of tumor tissue, and a lower value is conversely associated with non-malignant tissue. The human raters were primed to outline the primary tumor only. In order to setup the machine learning similarly, we labelled all disconnected objects in space provided by the ML upon prediction of new patients, only preserving the largest object.

### Assignment of training and validation data

3D masks of the primary tumor aligned with the VIBE imaging data were outlined by two expert radiologists (rater 1 = J.A.D. with> 4 years of experience with pelvic imaging; rater 2 = K.S.W-L. with > 10 years of experience with pelvic imaging). Tissue associated with primary tumor was assigned a value of one, while remaining tissue was designated the value zero. The radiologists were blinded to each other’s segmentations. A total of n = 34 patients was outlined by both raters and is referred to as the test data set. The test data set was unseen by the training algorithm and used for an unbiased evaluation of segmentation performance after training was completed. A total number of 105 patients were outlined by either one of the radiologists (n$_\text {rater1}$ = 79, n$_\text {rater2}$ = 26). This data set was randomly split into a train (n = 84 patients) and a validation data set (n = 21), (in agreement with a train/validation split of 0.2). The validation data set was used to obtain an unbiased evaluation of the model fit on the training data set while tuning model hyperparameters. Each rater tumor volumes are referred to as $V_{R1}$ and $V_{R2}$ , while machine learning tumor volume is referred to as $V_{ML}$. The radiologists had access to VIBE, T2-weighted images, and DWI (diffusion weighted imaging) b1000 images when performing manual tumor delineation in order to imitate routine radiological work-up.

### Metrices for evaluation of segmentation performance

Segmentation performance was estimated for the test data set. We evaluated volumetric segmentation accuracy according to the Dice similarity coefficient (DSC), limiting to 0 $\le$ DSC $\le$ 1^27^. A high value of DSC indicates high performance of the segmentation. The Dice similarity coefficient is a volumetric measure for segmentation performance, which to a limited extent is sensitive to outliers. For this reason, we also computed the Hausdorff distance, defined as the greatest of all distances from a point in one set to the closest point in the other set. Hausdorff distances (HD) were computed using the python library function directed_hausdorff implemented in scipy. A low Hausdorff distance indicates high segmentation accuracy in terms of boundary similarity.

In order to map segmentation errors in the outer tumor boundaries, we also computed average surface distance (ASD) measured from the ground truth mask surface to the ML mask surface in the Euclidean sense^28^. A smaller value of ASD is associated with an improved segmentation.

Statistical analyses were performed in MATLAB with Statistics Toolbox Release 2018b and using R (https://www.r-project.org) for statistical computing. Statistical hypothesis testing was performed under a significance level of $\alpha$=0.05. Lilliefors goodness-of-fit test of composite normality (Lillietest in MATLAB) was used to determine whether sample data was drawn from a normally distributed population. Whenever non-normality data distribution, a non-parametric statistical test was applied. Assessment of consistency in log tumor volume estimates was performed with the intraclass correlation coefficient (ICC). Bland-Altman plots are shown to visualize agreement in tumor volumes. Limits of agreement (LoA) are calculated as the mean difference $+/- 1.96$ standard deviation of the difference. Box plots are used to visualize sample tumor volumes, Dice similarity coefficients and Hausdorff distances. Friedman test for multiple comparison was used to assess rank differences in tumor volume estimates between the raters and ML.

Differences in DSC, HD, and ASD between humans and machine were explored along Wilcoxon signed rank test. We computed a set of three agreements A, B, and C where A = rater 1 against rater 2, B = rater 1 against ML, and C = rater 2 against ML (cfr. Table 1). Individual differences in the three agreements were accessed by pairwise subtraction and testing for zero median applied to A-B and A-C.

## References

[1] Amant F (2005). Endometrial cancer. The Lancet.

[2] Salvesen HB, Haldorsen IS, Trovik J (2012). Markers for individualised therapy in endometrial carcinoma. Lancet Oncol..

[3] Fasmer KE (2018). Preoperative quantitative dynamic contrast-enhanced MRI and diffusion-weighted imaging predict aggressive disease in endometrial cancer. Acta Radiol..

[4] Ueno Y (2017). Endometrial carcinoma: MR imaging-based texture model for preoperative risk stratification-a preliminary analysis. Radiology.

[5] Wang T, Sun H, Guo Y, Zou L (2019). 18F-FDG PET/CT quantitative parameters and texture analysis effectively differentiate endometrial precancerous lesion and early-stage carcinoma. Mol. Imaging.

[6] Ytre-Hauge S (2018). Preoperative tumor texture analysis on MRI predicts high-risk disease and reduced survival in endometrial cancer. J. Magn. Reson. Imaging.

[7] Ytre-Hauge S (2015). Preoperative tumor size at MRI predicts deep myometrial invasion, lymph node metastases, and patient outcome in endometrial carcinomas. Int. J. Gynecol. Cancer.

[8] Krizhevsky, A. Sutskever, I. & Hinton, G. E. Imagenet classification with deep convolutional neural networks. In *Advances in Neural Information Processing Systems 25*, 1097–1105 (Curran Associates, Inc., 2012).

[9] Lundervold AS, Lundervold A (2019). An overview of deep learning in medical imaging focusing on MRI. Z. Med. Phys..

[10] Yamashita R, Nishio M, Do RKG, Togashi K (2018). Convolutional neural networks: an overview and application in radiology. Insights Imaging.

[11] Esteva A (2017). Dermatologist-level classification of skin cancer with deep neural networks. Nature.

[12] Haenssle H (2018). Man against machine: diagnostic performance of a deep learning convolutional neural network for dermoscopic melanoma recognition in comparison to 58 dermatologists. Ann. Oncol..

[13] Arbabshirani MR (2018). Advanced machine learning in action: identification of intracranial hemorrhage on computed tomography scans of the head with clinical workflow integration. NPJ Dig. Med..

[14] Steiner DF (2018). Impact of deep learning assistance on the histopathologic review of lymph nodes for metastatic breast cancer. Am. J. Surg. Pathol..

[15] Lu MT (2019). Deep learning to assess long-term mortality from chest radiographs. JAMA Netw. Open.

[16] Xie J, Liu R, Luttrell J, Zhang C (2019). Deep learning based analysis of histopathological images of breast cancer. Front. Genet..

[17] Ronneberger, O. Fischer, P. & Brox, T. U-net: convolutional networks for biomedical image segmentation. In *International Conference on Medical Image Computing and Computer-assisted Intervention*, 234–241 (Springer, 2015).

[18] Gibson E (2018). NiftyNet: a deep-learning platform for medical imaging. Comput. Methods Progr. Biomed..

[19] Kamnitsas K (2017). Efficient multi-scale 3D CNN with fully connected CRF for accurate brain lesion segmentation. Med. Image Anal..

[20] Pawlowski, N. *et al.* Dltk: state of the art reference implementations for deep learning on medical images. arXiv preprint arXiv:1711.06853 (2017).

[21] Çiçek, Ö. Abdulkadir, A. Lienkamp, S. S. Brox, T. & Ronneberger, O. 3D u-net: Learning dense volumetric segmentation from sparse annotation. In *Medical Image Computing and Computer-Assisted Intervention—MICCAI*, 424–432 (Springer International Publishing, 2016).

[22] Xia, Y. *et al.* 3d semi-supervised learning with uncertainty-aware multi-view co-training. *The IEEE Winter Conference on Applications of Computer Vision* **3646–3655** (2020).

[23] Zhou, Y. *et al.* Prior-aware neural network for partially-supervised multi-organ segmentation. In *Proceedings of the IEEE International Conference on Computer Vision* **10672–10681** (2019).

[24] Cox RW (2004). A (sort of) new image data format standard: Nifti-1: we 150. Neuroimage.

[25] Chollet, F. Keras. https://github.com/fchollet/keras (2015).

[26] Abadi, M. *et al.* TensorFlow: large-scale machine learning on heterogeneous systems (2015).

[27] Dice LR (1945). Measures of the amount of ecologic association between species. Ecology.

[28] Yeghiazaryan V, Voiculescu ID (2018). Family of boundary overlap metrics for the evaluation of medical image segmentation. J. Med. Imaging.

